# SARCOPENIA ACCORDING TO BRAZILIAN AND EWGSOP 2 CUTOFFS: DATA FROM SARCOS

**DOI:** 10.1093/geroni/igz038.3281

**Published:** 2019-11-08

**Authors:** Julia M Menezes, Angela T Paes, Alberto Frisoli

**Affiliations:** 1 Faculdade Israelita de Ciências da Saúde Albert Einstein, São Paulo, São Paulo, Brazil; 2 Universidade Federal de São Paulo, São Paulo, São Paulo, Brazil

## Abstract

Cutoff values for lean mass and muscle strength are still controversial in the diagnosis of sarcopenia. The use of European, American and Asian consensus outside these regions may lead to important diagnostic errors. We hypothesized that there are significant differences between the cutoff points from Brazil and Europe in older people. This is a cross-sectional analyses of 502 older adults from SARCOS study, conducted at São Paulo - Brazil. All subjects underwent DXA analyses of total body. Lean mass was obtained from appendicular lean mass by height2 and muscle strength by dynamometer of dominant hand. The Brazilian cutoff points were based on 25th percentile by gender. The European ones were from EWGSOP 2. Agreement was assessed by the Kappa coefficient. The mean age was 78.39 ± 7.08 years old and 277 (55.18%) individuals were women. Among the ethnic groups, 339 (67.53%) were caucasian, 145 (28.88%) afrodescendants and 18 (3.59%) asians. The Brazilian cutoffs for muscle strength were 26 kg for men and 16 kg for women (equivalent to EWGSOP2); while those for lean mass were significantly lower, 6.56 kg/m2 vs. 5.56 kg/m2, respectively. The prevalence by EWGSOP 2 was higher than that obtained by the Brazilian cutoff points (20.32% vs 14.14%, p <0.001), even though these criteria presented Kappa = 0.792; p <0.001. Considering these disparities, 6 out of 100 subjects are considered sarcopenic by European criteria and not by the Brazilian cutoffs. There are significant differences in sarcopenia cutoffs between Brazil and Europe, and this cause important diagnostic variations.

